# Aberrant branched-chain amino acid catabolism in cardiovascular diseases

**DOI:** 10.3389/fcvm.2022.965899

**Published:** 2022-07-15

**Authors:** Yixiao Xiong, Ling Jiang, Tao Li

**Affiliations:** ^1^Department of Anesthesiology, National-Local Joint Engineering Research Centre of Translational Medicine of Anesthesiology, West China Hospital of Sichuan University, Chengdu, China; ^2^Laboratory of Mitochondria and Metabolism, West China Hospital of Sichuan University, Chengdu, China

**Keywords:** branched-chain amino acids, catabolism, cardiovascular diseases, heart failure, coronary artery disease

## Abstract

Globally, cardiovascular diseases are the leading cause of death. Research has focused on the metabolism of carbohydrates, fatty acids, and amino acids to improve the prognosis of cardiovascular diseases. There are three types of branched-chain amino acids (BCAAs; valine, leucine, and isoleucine) required for protein homeostasis, energy balance, and signaling pathways. Increasing evidence has implicated BCAAs in the pathogenesis of multiple cardiovascular diseases. This review summarizes the biological origin, signal transduction pathways and function of BCAAs as well as their significance in cardiovascular diseases, including myocardial hypertrophy, heart failure, coronary artery disease, diabetic cardiomyopathy, dilated cardiomyopathy, arrhythmia and hypertension.

## Introduction

Approximately 17.9 million people die from cardiovascular diseases, representing 32% of global deaths (1). More attention should be given to elucidating the pathogenesis of the disease. As a high energy consuming organ, the heart is more sensitive to nutrient metabolism (2). Therefore, a metabolic defect can have a significant impact on cardiac health and disease development. Traditionally, fatty acids and glucose are the two main metabolic substrates of the heart (3). Recent studies also found that heart failure is associated with ketone body utilization, which functions as a compensatory mechanism in maintaining cardiac energy homeostasis (4, 5). Are there other nutrient ingredients involved in the pathogenesis of heart failure, such as amino acids? Branched-chain amino acids (BCAAs) are the most plentiful amino acids in proteins. They belong to the group of essential amino acids, which in animals are only present in small amounts. Aberrant BCAA homeostasis has been observed in a number of disorders, such as type 2 diabetes, liver cirrhosis, renal failure, and cancer (6–9). They present diverse biological functions in the pathogenesis of these diseases. BCAA metabolism has been shown to be effective in preventing or treating hepatic encephalopathy, reducing fatigue during exercise, promoting healing, and stimulating insulin production (10–12). Recently, the development of cardiovascular diseases has also been linked to elevated levels of BCAAs (13–15).

## Branched-chain amino acid synthesis, metabolism and catabolites

**FIGURE 1:**
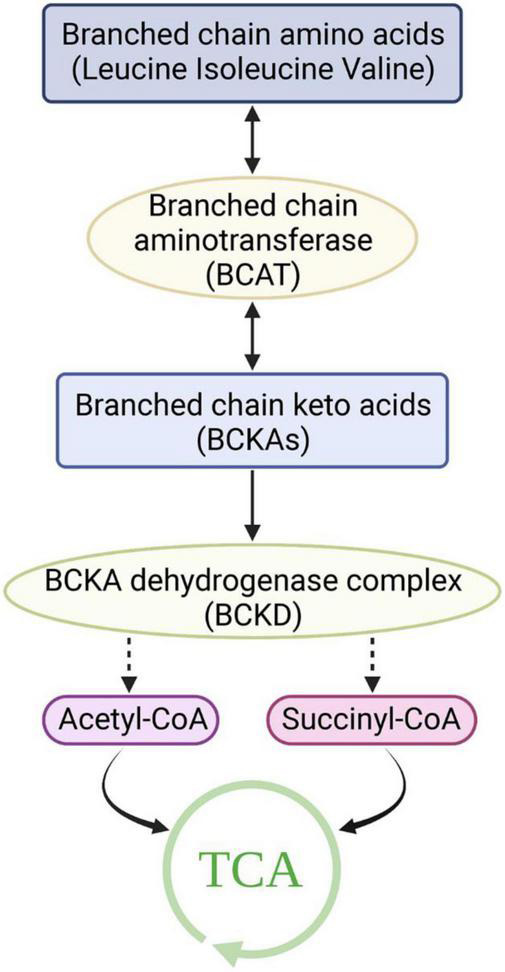
Catabolism of branched-chain amino acids. TCA, Tricarboxylic acid cycle.

Although BCAAs are essential amino acids that cannot be synthesized by animals, their synthesis occurs in bacteria, fungi, and plants (16). In these species, BCAAs are derived from the transamino precursor of valine, α-ketoisovaleric acid, which is synthesized by the same enzymes as valine and isoleucine (16). Pyruvate is the source of carbon in valine and leucine, while the carbon in isoleucine is derived from threonine. Unlike most amino acids, the first step of BCAA catabolism does not occur in the liver because branched-chain aminotransferases (BCATs) are the first enzymes in the BCAA catabolic pathway, which have low activity in the liver. In humans, BCAAs are primitively transaminated to form branched-chain α-keto acids (BCKAs) by BCATs (17). There are two genes that encode BCATs: BCAT1 and BCAT2. BCAT1 encodes a cytoplasmic protein and is mainly expressed in the brain, while BCAT2 encodes a mitochondrial protein (18, 19). The second catabolic enzyme of BCAAs, branched-chain α-ketoacid dehydrogenase (BCKDH), is a multienzyme complex located on the inner surface of the mitochondrial inner membrane that shares many of the same properties as the pyruvate dehydrogenase complex. Similar to the PDH complex, BCKDH catalyzes oxidative decarboxylation, releases carbon dioxide (CO2), and adds a coenzyme a (CoA) moiety to the oxidized BCKA product. Branched acyl-CoA ester is generated through irreversible decarboxylation of BCKA. BCKDH is regulated by phosphorylation and dephosphorylation. Specific kinase-mediated phosphorylation leads to inactivation, and specific phosphatase-mediated dephosphorylation activates the enzyme (20–22). A mitochondrial-targeted type 2c serine/threonine protein phosphatase, PP2Cm, has been identified as a key phosphatase of BCKDH and plays a critical role in regulating BCAA catabolism and homeostasis. BCKDH kinase is allosterically inhibited by BCKAs, whose maximal affinity is for α-ketoisocaproic acid (α-KIC), allowing the elevation of BCKAs to promote their own oxidation (23). Eventually, the carbons of BCAAs are either lost as carbon dioxide or enter the tricarboxylic acid cycle (Figure 1).

Albeit that BCAAs are catabolized in mitochondria, the catabolic intermediates in this process are not trapped in the mitochondrial matrix. For example, 3-hydroxyisobutyric acid (3-HIB), which is part of the valine catabolic pathway, is secreted from muscle cells into plasma, activates endothelial fatty acid transport, stimulates muscle fatty acid uptake *in vivo* and promotes lipid accumulation in muscle, leading to insulin resistance in mice (24). Meanwhile, one of the leucine oxidation products, acetoacetate, can be detached from the matrix prior to ketone oxidation. Before being oxidized by BCKDH, the α-carbon of α-keto acids can be reduced to generate branched α-hydroxy keto acids, while a small fraction of α-KIC can also be converted to beta-hydroxy-beta-methylbutyrate (HMB) by cytoplasmic dioxygenases (25, 26). BCAAs also facilitate the synthesis of several distinctive lipids, ranging from n-acyl amino acids to branched-chain fatty acids and odd-chain fatty acids (27).

## Branched-chain amino acid-regulated signaling pathways

### mTOR

In addition to serving as energy substrates, BCAAs and their metabolites play a critical role in the body in metabolic regulation and signal transduction. The regulation of leucine on the targets of the mTOR pathway is the most intensively studied area (28–30). Leucine activates mTORC1, a key growth regulator, and controls a number of cellular processes, including protein synthesis and cell growth (31–33). mTORC1 is signaled by amino acids through Rag guanosine triphosphatases (GTPases). GATOR1 and GATOR2 regulate Rags, and sestrin2 (a GATOR2-interacting protein) inhibits mTORC1 signaling (33). Therefore, leucine activation of mTORC1 in cells requires Sestrin2, which suggests that Sestrin2 is a leucine sensor of the mTORC1 signaling pathway (33, 34). Notably, a small GTPase called SAR1B was recently found to bind to leucine and activate mTORC1 through conformational changes (35). Meanwhile, both glutamate dehydrogenase and valine metabolites are involved in several signaling pathways (24, 36, 37). BCAAs activate mTOR in various metabolic responses. For example, mTOR activation also triggers metabolic changes in tissues, such as muscle and liver, by altering insulin sensitivity (38–42). BCAAs and BCKAs can also inhibit pyruvate and fatty acids in transport and utilization (39, 43).

### Glutamate dehydrogenase

**FIGURE 2:**
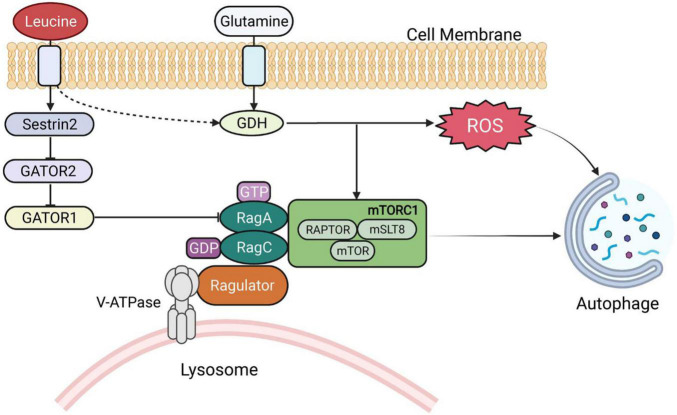
BCAA-regulated signaling pathways. GDH, glutamate dehydrogenase; RAG, RAS-related GTP-binding proteins; ROS, reactive oxygen species.

Leucine is a poor substrate for glutamate dehydrogenase (GDH) and is a metabolic activator of the enzyme. A dual mechanism for GDH flux regulation of autophagy was identified, both by delivering cellular amino acid availability to MTORC1 and by generating reduced equivalents that interfere with reactive oxygen species (ROS) accumulation (Figure 2) (44). Meanwhile, in low-glucose states, leucine and α-KIC are strong insulin secretagogues. In contrast, leucine stimulates insulin release by activating glutamate dehydrogenase, and α-KG is formed by the oxidative deamination of glutamate by GDH (45). Protein meal-induced hypoglycemia, hyperinsulinemia and hyperammonia are symptoms caused by GDH mutations resulting in leucine hyperactivation (by reducing GTP inhibition) (46). Additionally, α-KIC functions as a strong insulin secretagogue, in part through its transamination, which generates both leucine to activate GDH and α-KG to enter the TCA cycle (47).

## Branched-chain amino acids in risk factors related to cardiovascular diseases

### Inflammation

Chronic inflammation has a pivotal role in cardiovascular diseases, and it is both a marker before the onset of heart failure with preserved ejection fraction (HFpEF) and a factor in the death of HFpEF (48, 49). Obesity, also a cardiovascular risk factor, can lead to systemic inflammation in the body and then promote macrophage release of proinflammatory cytokines to infiltrate adipose tissue (50, 51). The role of BCAA catabolism in adipogenesis and resistance to adipocyte inflammation has been elucidated; however, the role of BCAA catabolism in macrophage function is unclear (52, 53). In a recent article, it was mentioned that increased uptake of leucine was found after stimulation of the RAW264.7 mouse macrophage cell line using lipopolysaccharide under normal oxygen supply and hypoxic conditions, suggesting that LPS stimulation of macrophages leads to an increase in BCAAs as alternative carbon sources for glucose and glutamine (54). Another article showed significant anti-inflammatory effects of both acute and chronic BCAA supplementation and highlighted the potential role of isoleucine, one of the BCAAs, in modulating the immune profile of macrophages prior to LPS stimulation (55). Macrophage BCAT1, which interferes with metabolic reprogramming, has also been suggested as an attractive therapeutic target for chronic inflammatory diseases (56). All these results demonstrate that although the exact mechanism is unknown, BCAA metabolites and enzymes in their metabolic pathways may be involved in systemic inflammatory cardiovascular diseases such as HFpEF by causing chronic inflammation in non-cardiac cells such as adipocytes and immune cells.

### Aging

The incidence of cardiovascular diseases increases significantly with aging. Downregulation of BCAT1 was found to be a highly significant feature in aged mice (57). A recent clinical trial also suggests that continuous BCAA supplementation may be associated with improved poor nutritional status in elderly patients and that specific BCAA supplementation may also enhance cognitive performance as mitochondrial function improves (58). Meanwhile, several studies have shown that weakness produced by aging is associated with low blood BCAA levels and changes in other amino acids (59, 60). In one study, BCAA consumption was positively correlated with leukocyte telomere length in middle age but negatively correlated with frailty in old age (61). It has been shown that long-term dietary BCAA manipulation impacts lifespan in mice by regulating food intake in a way that involves interactions with other amino acids, such as tryptophan and threonine (30). Although BCAAs have been shown to be associated with aging in many articles and clinical trials, the specific mechanisms involved in aging and the pharmacological targets for exerting interventions are still unclear, and further studies are needed.

### Obesity

Obese patients with disorders of glucolipid metabolism have also been found to have atrial and ventricular remodeling (62–64). People with obesity have larger left ventricle dimensions, partly due to both an increased intravascular volume and altered LV filling properties (65). A metabolomic analysis of plasma from obese and lean populations showed abnormal BCAA catabolism and increased plasma BCAA levels in obese people, and this phenomenon is associated with insulin resistance due to obesity (29). Studies have shown that BCAAs are closely associated with abnormalities in glucose and lipid metabolism, but the underlying mechanisms are poorly understood (15, 66, 67). Meanwhile, in brown adipose tissue (BAT), cold stimuli enhance mitochondrial BCAA uptake and oxidation, which leads to enhanced BCAA clearance in the circulation, and in turn, defective BCAA catabolism in BAT results in defective BCAA clearance and thermogenesis, leading to the development of diet-induced obesity and glucose intolerance (68, 69). It was suggested that impaired BAT activity reduced systemic BCAA clearance in the presence of obesity or diabetes, while active BAT served as an important metabolic filter for circulating BCAAs, protecting the body from obesity and insulin resistance (70). However, the specific pathways between abnormal BCAA metabolism and obesity and their possible targets of intervention need further study.

### Diabetes mellitus

Diabetes is one of the major risk factors for cardiovascular diseases, and its cause of heart disease is the leading cause of death in diabetic patients. A series of observational studies have shown that elevated levels of circulating BCAAs *in vivo* are significantly associated with poor metabolism (71–75). It has long been documented that leucine seems to have direct effects on hypothalamic and brainstem processes involved in satiety (76). Several articles have also reported that BCAAs regulate the release of hormones such as leptin, GLP1 and gastrin, which may influence food intake and glucagon levels (77–79). Supplementation of BCAAs to cultured muscle cells resulted in activation of mTOR, impaired insulin-stimulated Akt/protein kinase B phosphorylation and reduced insulin-stimulated glucose uptake (74, 80). In clinical studies, elevated blood levels of BCAAs were positively correlated with insulin resistance and HbA1c levels (81, 82). Several longitudinal studies in different cohorts reported that elevated blood levels of BCAAs predicted future insulin resistance or type 2 diabetes mellitus (T2DM) (73, 83). Meanwhile, genetic analysis suggests that elevated plasma levels of BCAAs are associated with an increased risk of developing T2DM (84, 85). This raises another point that elevated BCAAs may be the result of insulin resistance, but it is also possible that elevated BCAAs may in turn cause diabetes through insulin resistance (85). As with diabetes, almost all cardiovascular diseases, such as heart failure and coronary heart diseases, have varying degrees of metabolic disorders, and the role of insulin resistance in cardiovascular diseases has long been reported (86, 87). It is certain that BCAA metabolic abnormalities do exist in obese patients, but whether BCAAs are involved in the altered vascular structure and metabolic disorders present in obese patients needs further elucidation.

## Branched-chain amino acids in cardiovascular diseases

### Heart failure

As one of the most common cardiovascular diseases, heart failure is a threat to human health. According to the reduction of ejection fraction, heart failure can be divided into heart failure with reduced ejection fraction (HFrEF) and heart failure with preserved ejection fraction (HFpEF). The development of heart failure is associated with major changes in myocardial metabolism. Overexpression of Kruppel-like factor 15 in heart failure inhibits BCAA catabolism and leads to accumulation of BCKAs in the myocardium, which can directly impact mitochondrial function and cellular viability (13). The early stages of heart failure are characterized by compensatory ventricular hypertrophy in response to increased hemodynamic stress, which is also associated with increased circulating BCAAs and BCKAs in humans and animal models (13, 88, 89). Prior diversion of BCKA to the reamination pathway may contribute to the constitutively high rate of protein synthesis, leading to myocardial hypertrophy and poor structural remodeling (21). Investigation of changes in the cardiac phosphorylation proteome after exposure to elevated BCKAs showed that chronic increases in BCKA could lead to the development of pathologic cardiac hypertrophy and impaired cardiac contractility (21). Meanwhile, the gene expression of PP2Cm, a key regulator of BCAA catabolism, is reduced in hypertrophic hearts and further reduced in failing hearts (87). Mouse models carrying the genetically inactivated PP2Cm-encoding gene ppm1k (PP2Cm-KO) show a further decline in cardiac function with increasing age when compared to wild-type mice (90). At the same time, eight weeks after transverse aortic constriction (TAC), PP2CM-KO mice showed a significant reduction in left ventricular ejection fraction, ventricular dilatation, and elevated wet lung weight (90). Abnormal BCAA metabolism can lead to myocardial hypertrophy through insulin resistance, and ventricular remodeling due to myocardial hypertrophy can induce heart failure (91). After upregulation of BCKDH activity with the branched-chain α-keto acid dehydrogenase kinase inhibitor BT2, a reduction in systolic dysfunction and myocardial insulin resistance present in HFrEF was observed along with enhanced BCAA oxidation and reduced accumulation of BCAAs and BCAAs in the heart (13, 92). The role of BCAAs in the pathogenesis of HFpEF, for which no definitive treatment is available, has not been elucidated. However, it is worth noting the metabolism of non-cardiomyocytes, such as macrophages, in recent studies showing that macrophages cause fibrosis and diastolic dysfunction in HFpEF (93–95). Meanwhile, the enzyme BCAT, which initiates BCAA catabolism, regulates macrophage metabolic reprogramming, and the mitochondrial oxidative stress generated by inhibition of BCAA activity may lead to downregulation of metabolites between citrate and succinate in the tricarboxylic acid cycle (56, 96). The role of myocardial and non-myocardial BCAA metabolic pathways in the pathogenesis of heart failure deserves further investigation.

### Coronary artery disease

Coronary artery disease (CAD) is the most common cardiovascular disease (97). Although glucose and fatty acid metabolism have been recognized as core CAD mechanisms (98), scientists have demonstrated an independent relationship between elevated BCAA levels and the risk of CAD, regardless of the nature of the observed mechanism behind the elevated BCAA levels. The association between BCAAs and the risk of coronary heart disease remained significant after adjusting for traditional risk factors for coronary heart disease (99–101). The metabolism of the heart is dominated by fatty acid and glucose metabolism, and the heart consumes much less BCAA than other organs, so it is unlikely that a decrease in cardiac BCAA catabolism alone leads to an increase in plasma BCAAs (102–104). However, inhibition of systemic BCAA catabolism by knocking down the PP2Cm gene leads to elevated circulating and cardiac BCAA levels, which can compete with and inhibit gluconeogenesis in the heart via a non-transcriptional mechanism and exacerbate the cardiac response to ischemia/reperfusion (I/R) injury (13, 14). A recent discovery showed that BCAA/BCKA enhanced cardiac fatty acid oxidation levels by transcriptionally upregulating PPAR-α expression, thereby exacerbating lipid peroxidation toxicity and cardiac vulnerability to I/R injury (15). In the postinfarct heart, cardiac BCAA catabolism is impaired, resulting in myocardial BCAA accumulation; then, BCAAs activate myocardial mTOR signaling and subsequently contribute to cardiac dysfunction and remodeling following myocardial infarction (MI) (105). The metabolites of valine, one of the BCAAs, α-ketoisovaleric acid and propionyl-CoA show stronger effects on platelet activation than other BCAA metabolites, and propionyl-CoA is a key mediator of the BCAA metabolic pathway that mediates platelet activation. Excessive platelet activation can lead to microthrombosis, which can cause myocardial ischemia and infarction (106, 107). Additionally, dietary BCAA supplementation can not only facilitate platelet activation and increase thrombosis risk but also worsen contractility and increase infarct size following myocardial infarction (105, 106). These results reveal that abnormal branched-chain amino acid catabolism plays a crucial role in CAD (both MI and I/R), and the major signaling pathway mTOR and some of its intermediate metabolites are involved in myocardial metabolic reprogramming, leading to ventricular remodeling.

### Diabetic cardiomyopathy

Diabetic cardiomyopathy is a disease of the heart muscle that cannot be explained by hypertension, coronary artery atherosclerotic heart disease, or other heart diseases. A report found decreased BCAA metabolizing enzyme activity in myocardial tissue of mice with diabetic cardiomyopathy, suggesting abnormal BCAA catabolism (108). More importantly, cardiac ischemia–reperfusion injury with enhanced fatty acid oxidation was ameliorated after silencing of the PPARα pathway in mice with impaired BCAA metabolism, suggesting that PPARα may be a downstream pathway of BCAA metabolism leading to diabetic cardiomyopathy (15). A series of studies have suggested significant activation of the leucine-directed mTOR pathway in type 2 diabetic cardiomyopathy, and activation of the pathway also leads to further myocardial injury by inducing cellular autophagy and apoptosis (109, 110). However, autophagy is enhanced in type 1 diabetes but inhibited in type 2 diabetes, implying that the involvement of BCAA catabolism in diabetic cardiomyopathy cannot be simply generalized.

### Dilated cardiomyopathy

Dilated cardiomyopathy (DCM) is a primary myocardial disease of undetermined cause. It is characterized by left or right ventricular or bilateral ventricular enlargement with reduced ventricular contraction (111). A recent article analyzing specimens from patients with DCM found that inhibition of the lysosomal autophagy pathway was associated with the mTOR pathway, while metabolic analysis revealed a significant increase in valine and leucine in DCM hearts and a significant decrease in the levels of the protein phosphatase PP2Cm (112). An article suggests that embryonic mice lacking the mTOR pathway have significant developmental defects in the myocardium and can rapidly lead to dilated cardiomyopathy (113). However, the specific mechanism of BCAA metabolism involved in DCM is still unclear.

### Arrhythmia

A recent article reveals that the mechanisms by which plasma BCAAs content increased in mice contribute to the pro-arrhythmic state are associated not only with genetic BCAT2 deficiency, but also with acquired metabolic disorders such as diabetes, obesity and heart failure in which BCAA metabolism is impaired (114). In addition, when cardiomyocytes derived from human pluripotent stem cells were exposed to a high BCAAs environment, they also developed calcium dysregulation and arrhythmias similar to those in mice (114). A metabolic analysis of plasma samples from patients with cardiovascular disease and a prospective cohort study suggest that a significant correlation between elevated plasma BCAA levels and the occurrence of arrhythmias and strokes (115, 116). However, the specific pathways and genes involved in BCAA metabolic abnormalities leading to arrhythmias have not yet been identified, and more clinical evidence is warranted to validate this BCAA-associated phenotype.

### Hypertension

Hypertension is one of the most important risk factors for cardiovascular diseases and a key factor in the damage to blood vessels. Several cohort studies have shown that higher BCAA intake, in particular valine intake, is associated with a higher risk of incident hypertension (117–120). However, due to the diverse pathogenesis of hypertension, the exact mechanism of BCAAs in the development and progression of hypertension has not yet been elucidated, and further investigation is needed to unravel the complexity behind the circulating concentrations of BCAAs.

## Conclusion and perspective

**FIGURE 3:**
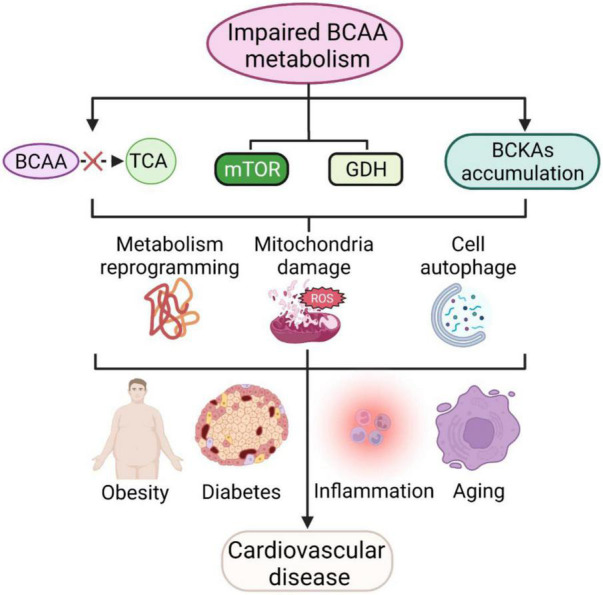
BCAAs and cardiovascular diseases. Impaired BCAA metabolism leads to less BCAA translocation into the TCA, activating mTOR and GDH pathways and accumulating BCKAs and further leads to metabolic reprogramming, mitochondrial damage and cellular autophagy. These processes are also involved in cellular inflammation, aging, obesity and diabetes mellitus, and further contribute to cardiovascular disease.

Branched-chain amino acids (BCAAs) and their metabolites can affect a variety of cellular processes, such as cell growth, protein synthesis, glucose metabolism and lipid metabolism. When BCAA catabolism is impaired, the oxidation of BCAA produces less acetyl coenzyme A and succinyl coenzyme A into the tricarboxylic acid cycle, while the mTOR complex and GDH are continuously activated, which, together with the accumulation of intermediate metabolites BCKAs, can further lead to metabolic reprogramming and reactive oxygen species production, resulting in mitochondrial damage and cellular autophagy. The above processes can not only be directly involved in cardiovascular diseases but also indirectly contribute to cardiovascular diseases by exacerbating systemic chronic inflammation, obesity, aging, diabetes and other cardiovascular disease risk factors (Figure 3). BCAAs have therapeutic potential, yet many controversies remain in the clinical application of BCAAs, and careful studies are needed to elucidate the effectiveness of BCAAs in most indications. Future goals include clarifying the specific mechanisms and therapeutic targets of BCAA involvement in cardiovascular disease and individualizing treatment based on specific patient characteristics.

## Author contributions

YX and LJ wrote the manuscript, figure legends, and created the figures. TL revised the manuscript. All authors contributed to the article and approved the submitted version.

## Conflict of Interest

The authors declare that the research was conducted in the absence of any commercial or financial relationships that could be construed as a potential conflict of interest.

## Publisher’s Note

All claims expressed in this article are solely those of the authors and do not necessarily represent those of their affiliated organizations, or those of the publisher, the editors and the reviewers. Any product that may be evaluated in this article, or claim that may be made by its manufacturer, is not guaranteed or endorsed by the publisher.
